# Serviceability and ductility behaviour of hybrid reinforced concrete beams partially composed of engineered cementitious composite (ECC)

**DOI:** 10.1038/s41598-025-15779-y

**Published:** 2025-08-21

**Authors:** Ahmed A. Radwan, Ahmed Ghallab, Ahmed M. Farghal Maree

**Affiliations:** Department of Structural Engineering, Ain Shams University, Cairo, Egypt

**Keywords:** Glass fiber reinforced polymers (GFRP), Hybrid bars (Steel and GFRP), Composed beams, Engineered cementitious composite (ECC), Flexural behaviour, Hybrid beams, Permanent formwork, Civil engineering, Structural materials

## Abstract

GFRP bars can be used partially or fully instead of steel bars in RC-beams to eliminate corrosion problems, especially in harsh environments. Also, the implementation of the combined use of engineered cementitious composite (ECC) and the hybrid (steel-GFRP) reinforcement in concrete beams can enhance strength and serviceability. In this paper, seven beams; one as a control beam cast with traditional concrete, and six partial ECC RC-beams with GFRP bars only or RC-hybrid (steel-GFRP) bars were designed to investigate both deflection and ductility behaviour of such beam type. This research explored reinforcement types and two ECC configurations: (a) a bottom layer of varying thickness, (b) a U-shaped formwork. All specimens underwent four-point bending testing to examine cracks distribution, moment-strain, and moment-curvature relation, in addition to the evaluation of deflection equations and assessment of ductility of the composite beams. The experimental findings revealed that thicker ECC in the tension zone led to changes in the distribution of vertical cracks, stiffness, and energy absorption. The use of U-shaped ECC configuration generally leads to an increase in the ductility index. A specific construction technique with a corrugated surface was implemented, which created a roughened surface, improved the interface shear transfer, and eliminated bond failure at the interface. The deflection values calculated using ACI 318 -19 and CSA S806-12 equations showed good correlation with the experimentally measured deflections. In contrast, the ACI 440.1R-15 equations did not accurately capture the deflection behavior across all tested specimens.

## Introduction

Steel reinforcement corrosion poses a critical durability concern for reinforced concrete (RC) elements, particularly in aggressive environments like coastal zones, underground passages, and bridge substructures. This degradation mechanism can substantially reduce the functional lifespan of RC members^1^. The use of glass-fiber-reinforced-polymer (GFRP) bars provides numerous benefits in these types of members due to their superior advantages, such as corrosion resistance, less concrete cover, longer maintenance intervals, high chemical resistance, environmentally friendly, and non-corrosive as well as non-toxic.

Additionally, GFRP bars have a tensile strength almost three times greater than that of typical steel bars, which promotes them as a perfect choice for flexural reinforcement^2,3^. Contrary, GFRP bars have low tensile modulus compared to steel rebars and a linear elastic response up to rupture. Hence, the behavior of GFRP-RC-members is brittle rather than ductile, combined with large deflection and wide cracks. Consequently, the serviceability limit state is one of the main governing design limits for GFRP-RC beams^4–8^. Several codes, such as ACI440.1R-15^9^, CSA-S806-12^10^, JSCE-2001^11^, and ECP 208–2020^12^, specify the mode of failure of flexural GFRP-RC-members as over-reinforced failure mode with low ductility.

To overcome the above shortages, a hybrid reinforcement system composed of steel bars at the inner level of the tensile region and GFRP bars at corners placed in the same layer can be used^13,14^. This configuration enhances the reinforced concrete’s resistance to corrosion, improves its durability, reduces crack width and fracture spacing, and demonstrates higher stiffness after cracking^15–19^.

Also, to enhance the flexural and serviceability behavior of GFRP-members, the usage of engineered cementitious composite (ECC) materials with hybrid (steel-GFRP) reinforcement in concrete beams can be used. Previous studies show that ECC demonstrates exceptional tensile strain capacity, achieving (3–5) % elongation through the incorporation of polyethylene (PE) or polyvinyl alcohol (PVA) fibers. This enhanced ductility is achieved while maintaining fiber content below 2% by volume, optimizing both mechanical performance and material efficiency. The high strain range is caused by the sequential development of multiple cracks rather than the continuous increase in crack opening^20–27^. Ismail et al.^28^ investigated the influence of incorporating ECC in RC-beams containing different types of fibers and reinforced with steel bars only. In their experimental program, ECC was applied with a constant thickness either in the tensile or compressive zones of the beams. The findings demonstrated a significant enhancement in the flexural performance of the beams incorporating ECC when compared to those made entirely of conventional concrete. Batran et al.^29^ and Ismail et al.^30^ examined the same type of beams, substituting conventional concrete with lightweight concrete, and the study reported significant improvement in ductility and energy absorption. Also, ECC can enhance shear strength^31^ and be used as a strengthening material for RC-elements^32–37^.

### Research significance

Despite the research on ECC-composite RC-beams either with steel or GFRP, studies addressing hybrid reinforcement using both steel and GFRP in ECC-beams remain limited. Moreover, the use of U-shaped ECC formwork has not been sufficiently investigated in previous studies. Consequently, further work is required to better estimate the performance of these composed beams. To study the serviceability and the ductility of RC-hybrid-beams partially composed of ECC concrete, seven beams were experimentally tested. The main variables investigated were reinforcement type and ECC concrete configurations. ECC configurations were included as: (a) Tension layer only with different thickness, and (b) U-shaped formwork. The entire experimental program was carried out in the RC-unit in the Department of Structural Engineering at Ain Shams University^38^.

## Experimental program

An experimental investigation was conducted on seven partially ECC-RC-hybrid-beams with uniform cross-sectional dimensions (200 × 300 mm), incorporating varying combinations of steel and GFRP reinforcement. The specimens were subjected to two-point loading near midspan to evaluate how different reinforcement configurations influence serviceability and ductility performance.

### Material properties

Traditional concrete, ECC concrete, steel, and GFRP reinforcement were used in this testing program. For traditional concrete, the 28-day cylindrical compressive strength reaches 48 MPa. A 0.40 proportion of water to cement (W/c) was maintained in the concrete formulation. The mixture proportions with respect to the cement ratio are summarized in Table 1.

**Table 1:** Mixture proportions with respect to cement ratio of traditional Concrete.

Cement	Fine aggregate	Coarse aggregate	Water	Admixture (%)
1	1.70	2.7	0.40	0.50

For ECC, the 28-day cylindrical compressive strength reaches 40 MPa. A 0.23 proportion of water to binder (W/b) was maintained in the concrete formulation, where the binder includes both the weight of cement and fly ash. The mixture proportions with respect to the binder ratio of ECC concrete are presented in Table 2.

**Table 2:** Mixture proportions of ECC concrete with respect to binder ratio.

Cement	Flay ash	Fine aggregate	Water	HRWR admixture (%)	Fiber (Vol %)
0.45	0.55	0.36	0.23	0.59	2.00

HRWR, high range water reducer.

**Fig. 1:**
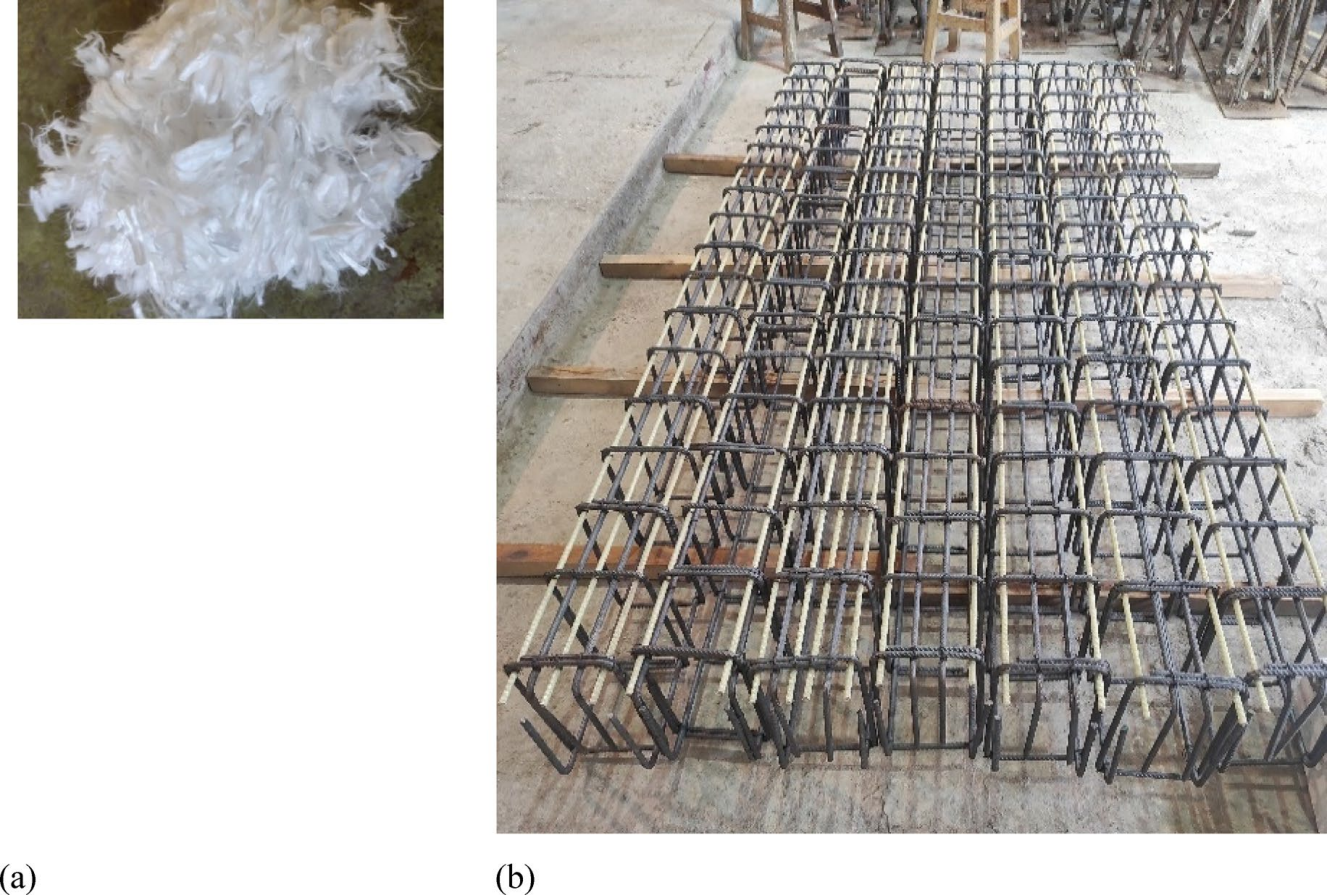
Constitutive material shape: (**a**) Polypropylene fibers; (**b**) Ribbed GFRP and steel bar cages.

Superplasticizer admixture, Sika viscoCrete-3425^39^ was used to reduce water content and enhance mix workability and self-compacting behavior. In the design of the ECC concrete mixture, Polypropylene fibers from Sika fiber^40^ with a volumetric friction of 2% were utilized. Table 3 shows the properties of the used fibers, while Fig. 1a shows the Polypropylene used fibers. In order to ensure equal distribution of polypropylene fibers in the mixture, fibers were introduced progressively while mixing was maintained for a period of 2 to 3 min.

**Table 3:** Properties of the used polypropylene fibers.

Length (mm)	Diameter (µm)	Aspect ratio (L/d)	Tensile strength (MPa)	Fiber elongation (%)	Specific gravity (kg/m^3^)
18	18	1000	300–400	> 80%	0.91

Deformed steel bar, Grade 500-T10 was used as the main longitudinal steel in compression and tension zones in all tested specimens. The diameter of the used GFRP bars was 10 mm with a fiber volume fraction of 73%, as specified by the manufacturer; however, the nominal diameter was 10.12 mm with a cross-sectional area of 80.43mm^2^. Figure 1b shows GFRP and steel bar cages for the tested beams. Standardized tensile testing was performed on longitudinal GFRP reinforcement following the specifications outlined in ASTM D7205^41^. The complete set of mechanical properties for both steel and GFRP reinforcing was tabulated in Table 4.

**Table 4:** Summary of mechanical tensile properties of steel and GFRP Bars.

Specimen No.	Area (mm^2^)	Yield stress F_y_ (Mpa)	Yield strain	Ultimate stress F_ult_ (Mpa)	Ultimate strain	Modules of elasticity E (GPa)
G500 Steel (T10)	78.5	576	0.00288	682	0.11296	200
GFRP (T10)	80.42	–	–	1000	0.02660	38

## Specimen design configuration

As shown in Table 5, the main properties of the experimental beams are detailed. The transverse reinforcement, which was uniform for all beams, consisted of T10-150 spacing, distributed with equal distance along the beam length to connect between ECC and traditional concrete as shear dowels. In this study, S and G stand for steel and GFRP reinforcement, respectively. The label 2G10-2S10 means the beam contains two 10 mm GFRP bars and two 10 mm steel bars. The letters C, E, and U specify the beam’s construction: C for conventional concrete, E for ECC in key areas, and U for U-shaped ECC formwork. The ending digits (50, 100, 150) indicate the ECC layer’s thickness (mm) in the tension region.

**Table 5:** Details of the tested beams.

Designation No.	Steel RFT (A_s_)	GFRP RFT (A_f_)	Formwork shape
2G10-2S10-C	2 T 10	2 T 10	﻿–
2G10-2S10-E50	2 T 10	2 T 10	(50 mm ECC bottom layer formwork)
2G10-2S10-E100	2 T 10	2 T 10	(100 mm ECC bottom layer formwork)
2G10-2S10-E150	2 T 10	2 T 10	(150 mm ECC bottom layer formwork)
4G10-E100	﻿–	4 T 10	(100 mm ECC bottom layer formwork)
4G10-EU50	﻿–	4 T 10	(U shape formwork)
2G10-2S10-EU50	2 T 10	2 T 10	(U shape formwork)

RFT, Reinforcement.

A_s_ is the steel reinforcement.

A_f_ is the GFRP reinforcement.

The tested beams were categorized into two distinct groups; the main investigated variables included reinforcement type and ECC concrete configurations. Table 6 shows the two categorized groups, parameters studied, and the beam designation numbers in each studied group.

**Fig. 2:**
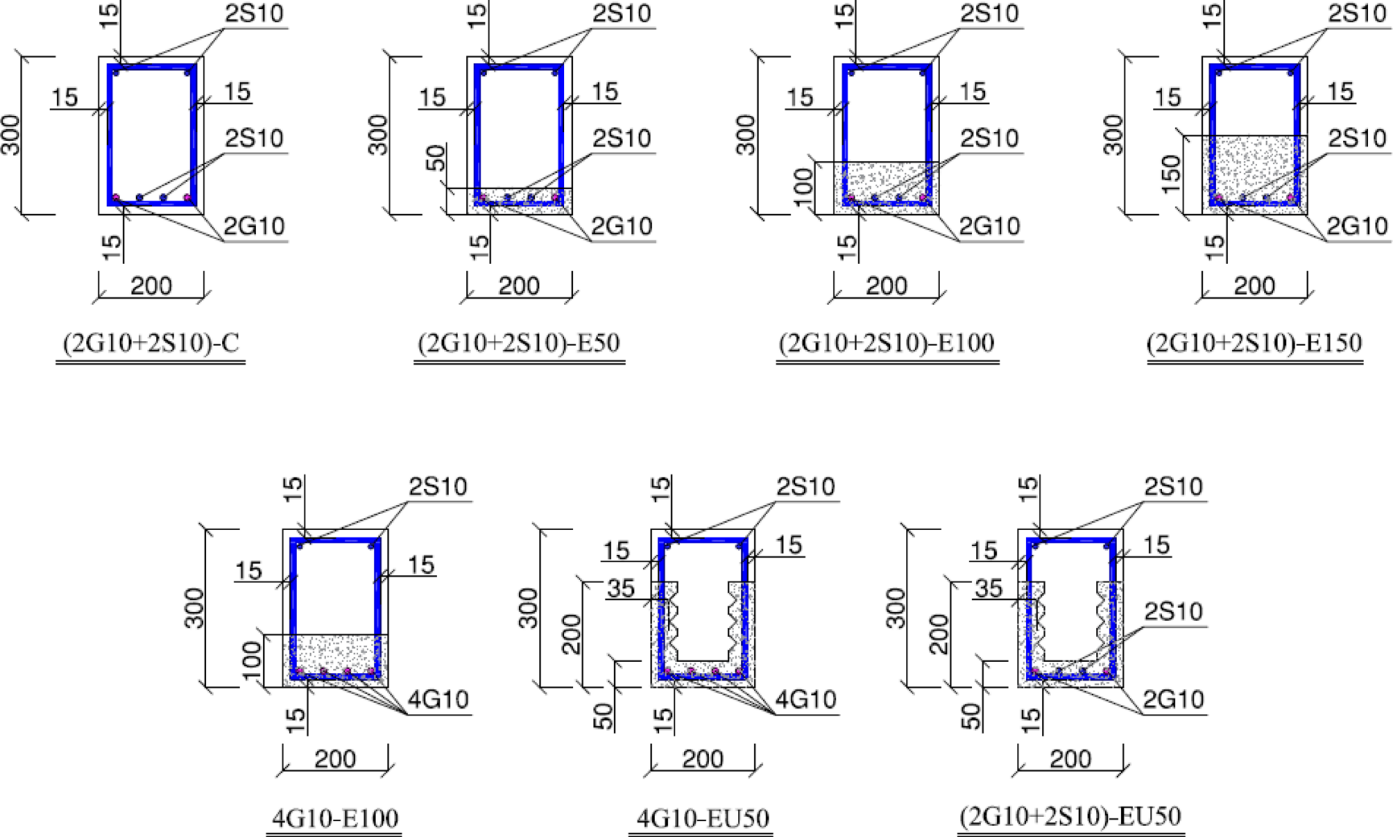

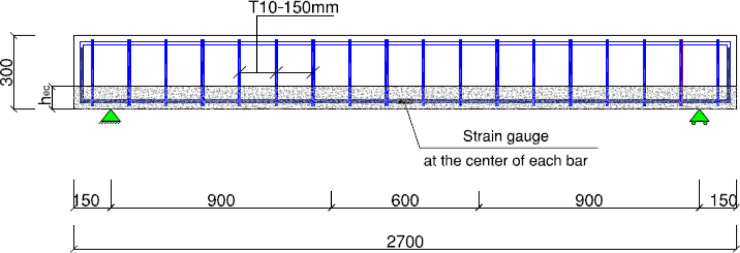
Reinforcement details of the tested beam.

- Group (1) studies the reinforcement type:
  Steel bars were used to replace a portion of the GFRP bars in the GFRP-RC-beams. This behaviour can be detected between (2G10 + 2S10-E100 & 4G10-E100) and (2G10 + 2S10-EU50 & 4G10-EU50).
- Group (2) study ECC concrete configurations:
  - effect of the thickness of the bottom ECC formwork, which checked by testing three beams with different thicknesses 50,100, and 150 mm, in the tension zone only. The height replacement ratio (denoted as r_h_) is defined as the proportion between the thickness of the ECC layer placed in the tension zone and the total depth of the tested beam. This ratio equals 0, 0.16, 0.33, and 0.50. A value of r_h_ = 0 indicates that the beam was cast entirely using conventional concrete without incorporating any ECC layer. This behaviour can be detected between (2G10 + 2S10-E50 & 2G10 + 2S10-E100 & 2G10 + 2S10-E150).
  - Shape of ECC formwork, which checked by comparing the flexural behaviour of beams having the same type of reinforcement, bar configuration, and the same amount of ECC but with different shapes. ECC is placed at the tension zone only with a thickness equal to 100 mm, that is almost 33% of the volume of the beam, or with U-shaped ECC that is cast in the tension zone and at each outer beam side. with the same volume ratio, 33%. This behaviour can be detected by comparing beams (2G10 + 2S10-E100 & 2G10 + 2S10-EU50) and (4G10-E100 & 4G10-EU50). The properties of the tested specimen are shown in Fig. 2.

**Table 6:** Designation number and studied parameters.

Group no.	Studied parameters	Designation no.
G1	Reinforcement type	(2G10 + 2S10-E100 & 4G10-E100) and (2G10 + 2S10-EU50 & 4G10-EU50)
G2	(a) ECC layer thickness (b) ECC U-Shaped	(a) (2G10 + 2S10-C & 2G10 + 2S10-E50 & 2G10 + 2S10-E100 & 2G10 + 2S10-E150) (b) (2G10 + 2S10-E100 & 2G10 + 2S10-EU50) and (4G10-E100 & 4G10-EU50)

**Fig. 3:**
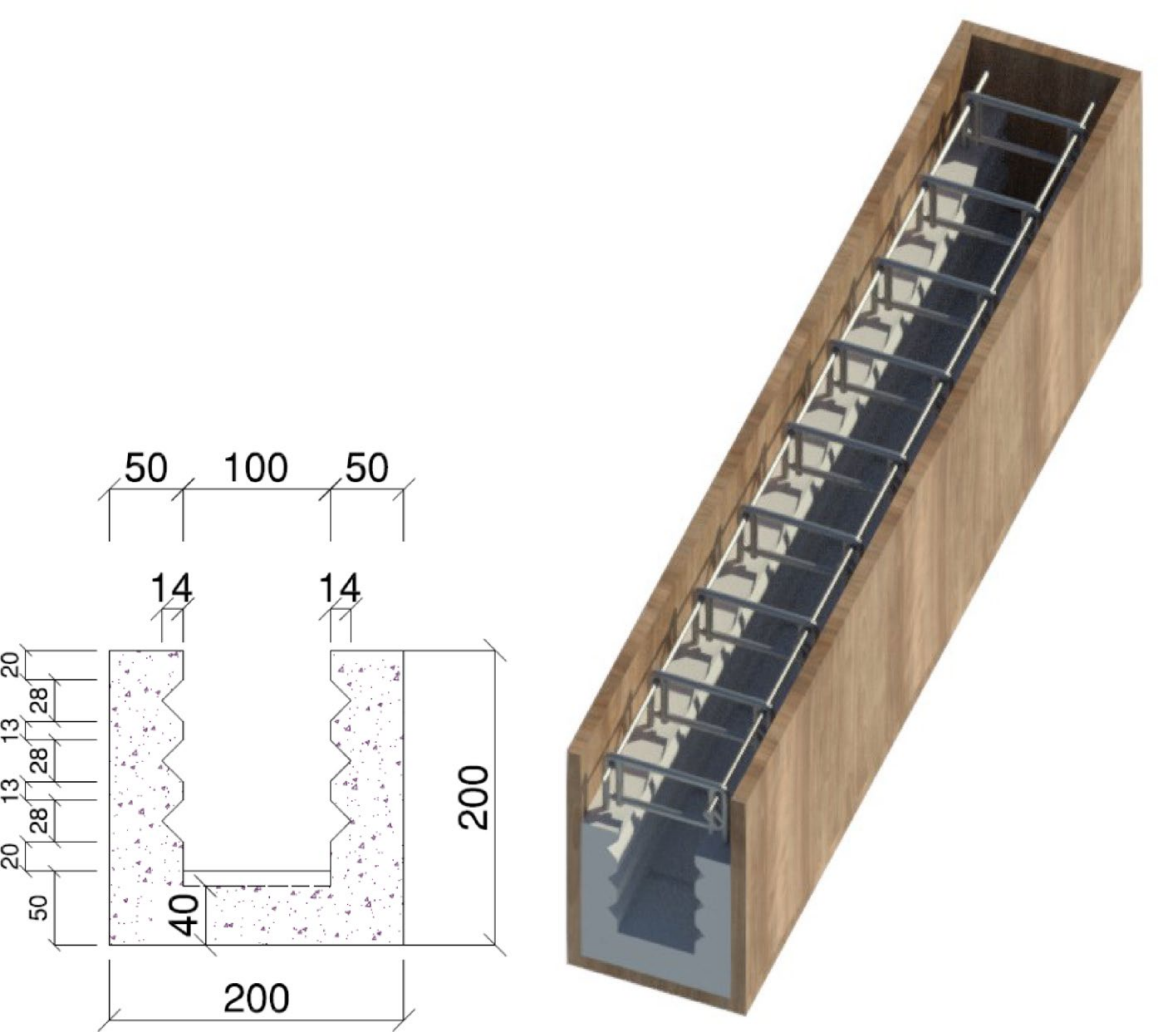
Cross-section dimension of used U-shape formwork.

Figure 3 shows the cross-sectional dimension of the used U-shape formwork and details of the reinforcement cage. The total height of the beam is 300 mm, and the U-shaped ECC portion was limited to 200 mm to allow it to function as a precast unit that can be integrated with other elements, such as a slab. The flexural reinforcement was embedded within the 50 mm-thick bottom ECC layer. Additionally, surface roughening was achieved by creating bottom grooves measuring 20 mm wide and 10 mm deep at 150 mm intervals, along with continuous side grooves along the full length of the beam. The casting process of beams 4G10-EU50 and (2G10 + 2S10)-EU50 began with preparing a 200 × 300 mm wooden formwork, into which a U-shaped reinforcement cage was placed. A specially designed inner U-shaped formwork was used to create shear keys and ensure a roughened surface for improved bond at the interface between ECC and traditional concrete. Notably, no reinforcement crossed this interface. The special formwork was then positioned inside the outer wooden mold, and ECC was cast to form the U-shaped section. After removing the inner formwork, the remaining section was cast using traditional concrete.

### Specimen preparation and casting

A two-stage casting method was adopted due to the differing concrete types. The ECC layers, varying in geometry and thickness, were poured first, followed by traditional concrete for the beam’s remainder. To optimize interfacial bonding, shear keys, dowels, and Addibond 65^42^ were employed alongside vibration for compaction. Post-casting, the surface was leveled to ensure planarity for testing. Full details of specimens’ preparation and casting can be found in^38^.

### Test setup and procedures

**Fig. 4:**
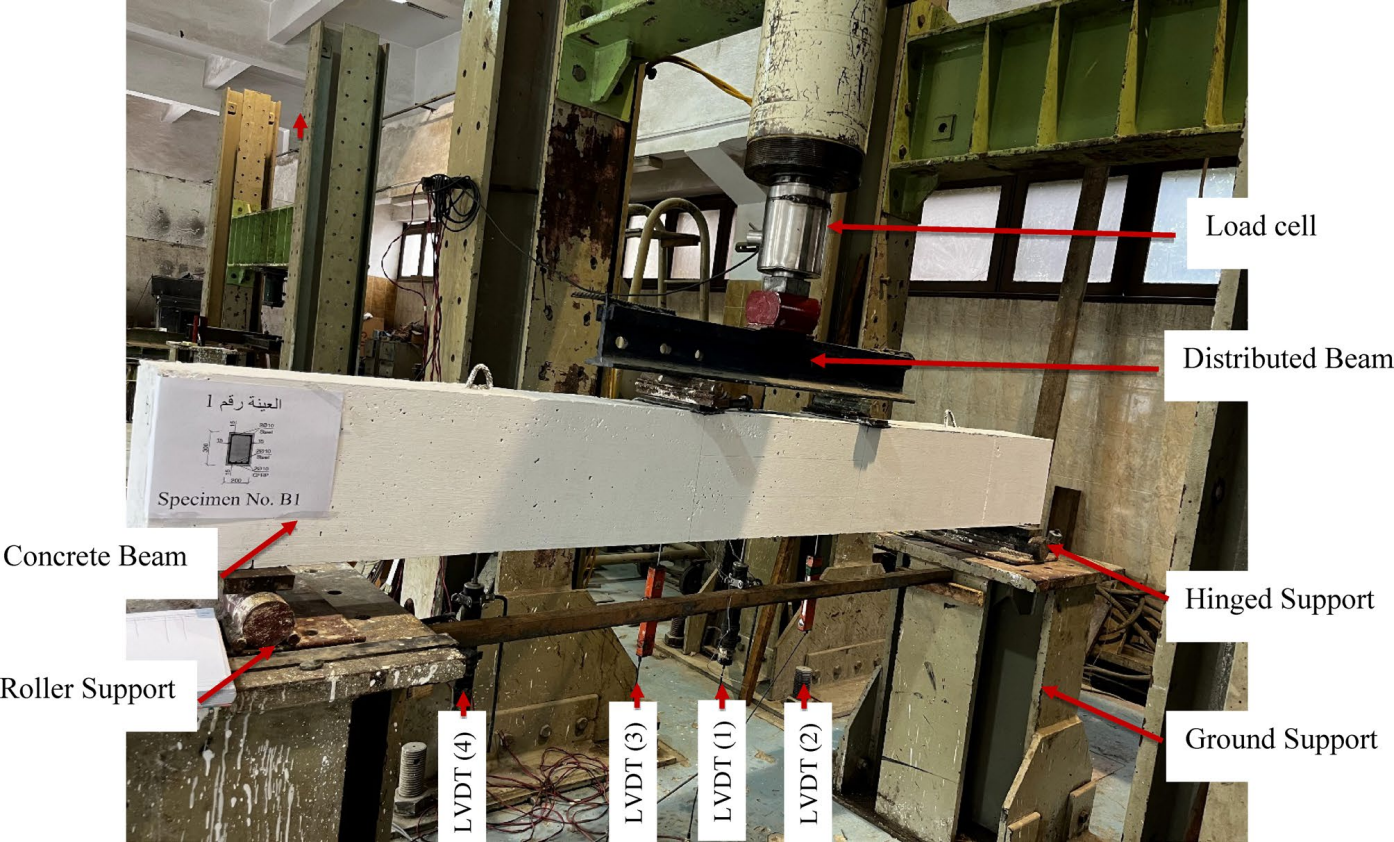
Typical test setup for the tested beams.

The experimental investigation employed a four-point bending test configuration for all beam specimens. Tensile strain monitoring was accomplished using four strain gauges, with two installed on GFRP reinforcement and two on steel bars. An additional strain gauge measured compressive strain in the concrete at mid-span, as presented in Fig. 4. Deflection monitoring utilized four LVDTs positioned at: (1) midspan, (2) under each loading point, and (3) at the midpoint between (loading points and supports). A load cell recorded applied loads, while all measurements (load, deflection, and strains) were automatically collected through a computerized data acquisition system.

The experimental configuration, illustrated in Fig. 4, employed a four-point bending test with a total span of 2400 mm between support centerlines. The loading points were spaced 600 mm apart, maintaining a (shear span/depth) ratio (a/h) of 3.0 to promote flexural failure mode. Loading was applied incrementally at 1 kN intervals, with strain and deflection measurements recorded at each load step until specimen failure.

## Experimental results and discussion

### Cracks distribution

As a general rule for RC-beams incorporating GFRP reinforcement and ECC, crack initiation typically occurred first in the ECC layer before propagating into the conventional concrete layer at mid-span and loading points. With increasing load, additional vertical cracks developed within the ECC region, while existing cracks in the traditional concrete exhibited progressive widening. The crack density in conventional concrete was notably lower compared to the ECC layer, with correspondingly larger crack spacings observed.

**Fig. 5:**
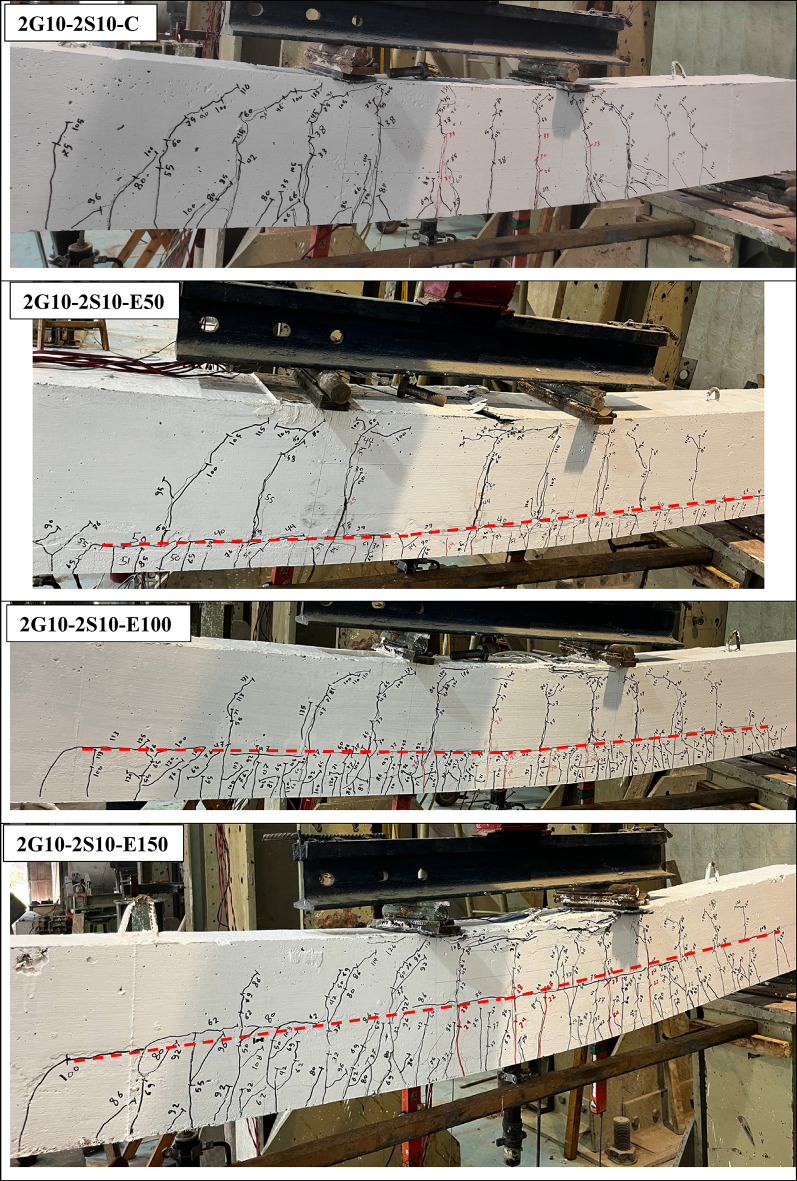

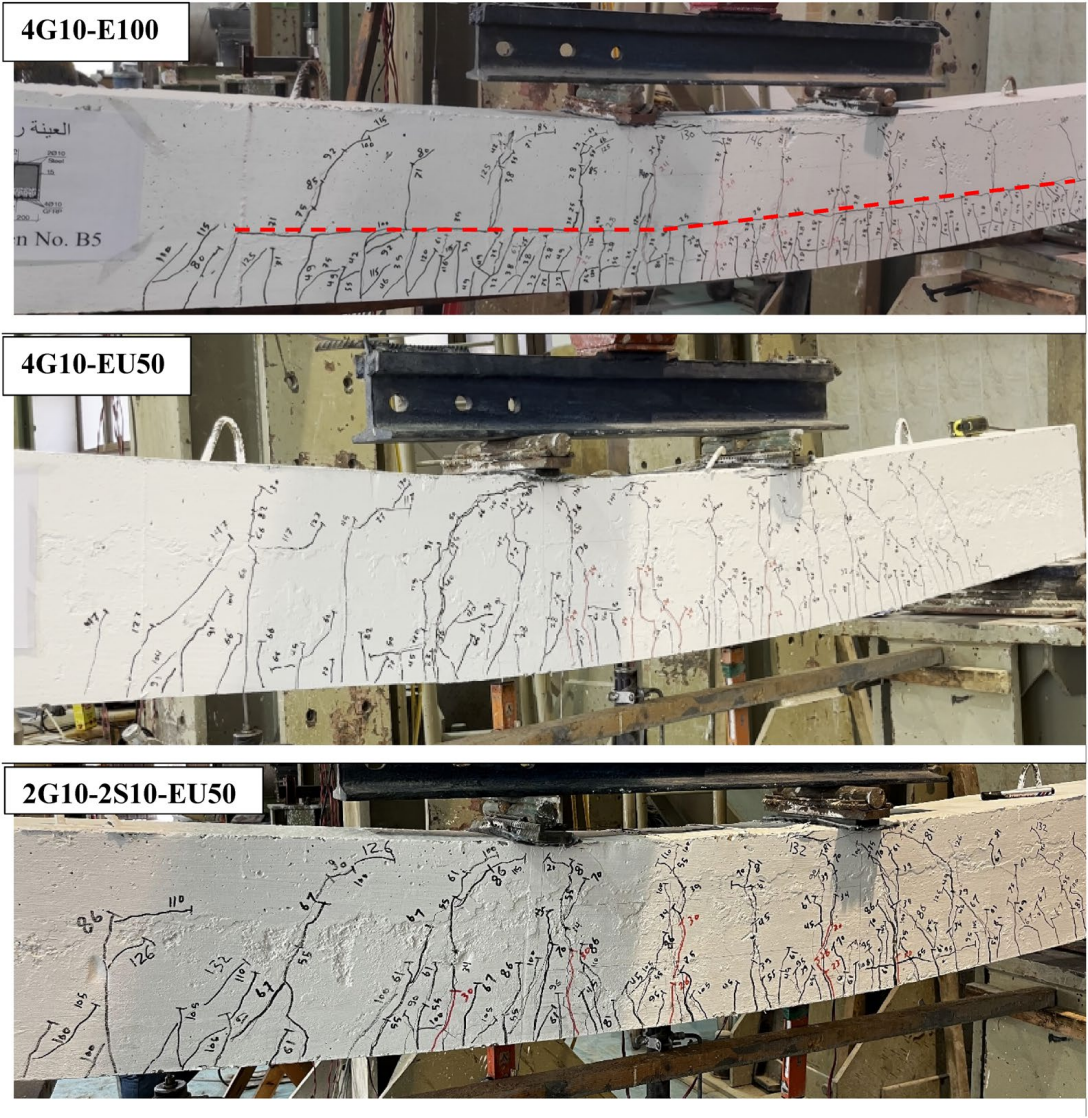
Cracks distribution for tested beams at failure.

Experimental observations from group (G1) specimens revealed that steel reinforcement effectively delayed the onset of initial cracking and interfacial horizontal cracks at the ECC-concrete interface, as presented in Fig. 5. G2 test results revealed that increasing the thickness of the bottom ECC layer further postponed horizontal crack formation. Notably, beams incorporating U-shaped ECC configurations exhibited no horizontal cracking throughout loading until failure, highlighting the superior performance of this geometrical configuration.

In addition, the experimental results demonstrated excellent bond integrity between the U-shaped ECC and core cast concrete, with no observed interfacial debonding. The U-shaped configuration significantly improved crack distribution patterns, offering enhanced protection for internal reinforcement against environmental exposure and corrosion. While all tested specimens exhibited vertical crack spacing of approximately 150 mm, U-shaped ECC beams showed reduced crack spacing below 100 mm. This improvement is attributed to the fiber-reinforced nature of ECC, which effectively controls crack propagation along the beam length.

The main advantage of using ECC becomes evident when comparing the crack width of specimen 2G10-2S10-C with those containing ECC. In the conventional specimen (2G10-2S10-C), cracks exhibited wider openings and greater spacing between successive cracks. In contrast, the ECC-enhanced specimens showed significantly narrower crack widths, with crack spacing generally not exceeding 50 mm. This behavior aligns with the fundamental concept behind incorporating fibers in ECC, which is to maintain tight crack widths while promoting the formation of multiple closely spaced cracks. This mechanism enhances the stiffness of the beam and improves its deflection performance.

The ultimate failure mechanism for all beam specimens was by compressive failure of the upper concrete region at mid span, accompanied by yielding of steel reinforcement, with no fracture observed in any steel bar. Both GFRP bars and ECC fibers maintained structural integrity throughout loading until concrete tensile failure occurred. Notably, shear failure modes were completely absent in all beams, as evidenced by the crack distribution patterns presented in Fig. 5.

### Load-deflection relationship

**Fig. 6:**
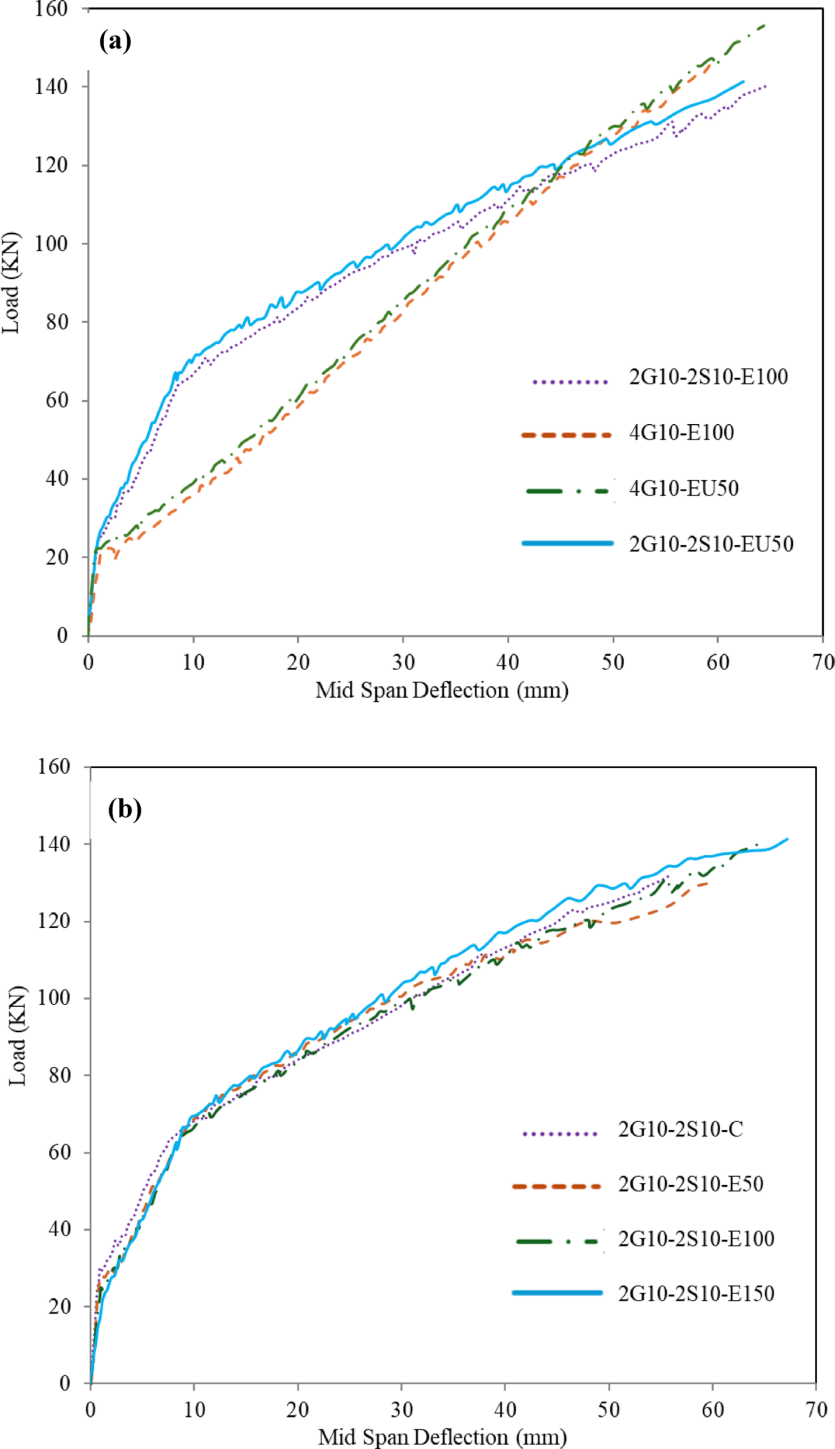
Load versus mid-span deflection relationships; G1 (**a**); G2 (**b**).

Figure 6 shows a relation between the experimental load in (KN) and mid-span deflection in (mm). For composite RC-hybrid beams, the load–deflection response can be characterized by three distinct stages. In the initial stage, the applied load is resisted primarily through the combined stiffness of the uncracked ECC layer and conventional concrete, with stress being effectively distributed among them. The second stage is marked by the initiation of micro-cracking in the tensile zone, particularly near midspan and beneath the loading points. This transition is typically reflected by the first inflection point on the load-deflection curve, where the stiffness begins to degrade due to crack formation within the ECC. The third stage, indicated by the second inflection point, corresponds to the onset of yielding in the tensile steel. This phase is accompanied by a significant increase in crack distribution within the ECC layer and a slight widening of pre-existing cracks in the surrounding conventional concrete, leading to further reduction in stiffness. At the end of this stage, the top concrete fiber reaches its strain capacity, and the beam failed resulting from compressive failure of the top concrete. In contrast, GFRP-beams exhibit a bilinear load-deflection behavior without a distinct yielding phase. According to G1, the presence of steel bars reduces the deflection values, where GFRP-RC beams get higher deflection values than hybrid RC-beams up to approximately 85% of the ultimate load, then the hybrid beams exhibited greater deflection values. According to G2, increasing the thickness of ECC led to an increase in the yield and ultimate deflection values. However, beams that have U-shaped ECC with the same reinforcement type get a slight reduction in deflection values during different loading stages. A comparison of deflection values between beam 2G10-2S10-EU50 and beam 2G10-2S10-E100 revealed reductions of approximately 25%, 8%, and 4% at the cracking, yielding, and ultimate load stages, respectively. A similar trend was observed when comparing beam 4G10-EU50 with 4G10-E100, where deflection decreased by about 88% at the cracking stage and 9% at the ultimate stage. More details regarding cracking, yielding, and ultimate load values in each stage can be found in^38^.

### Moment-strain relationship

**Fig. 7:**
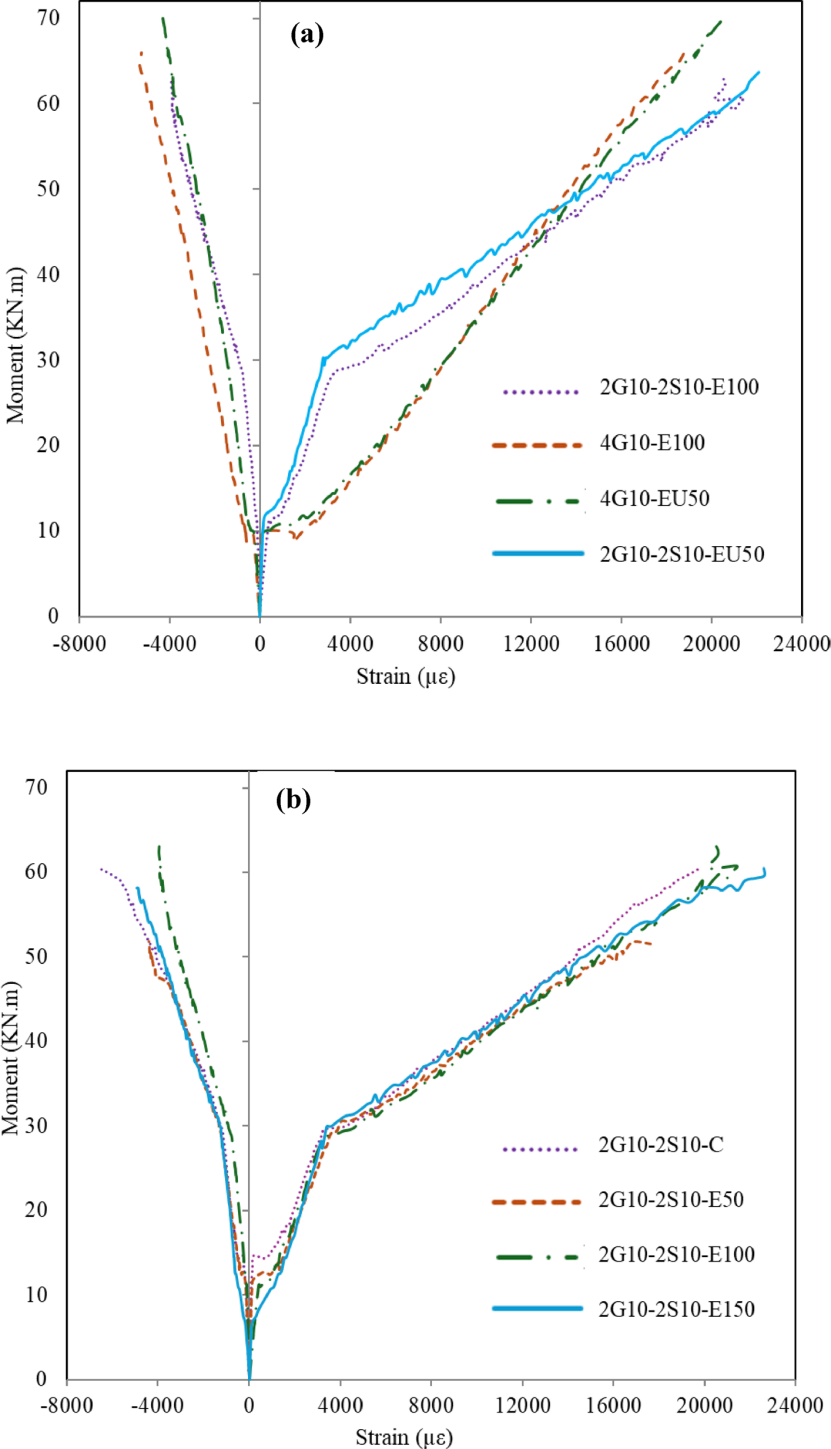
Moment versus concrete and GFRP strain relationships; G1 (**a**); G2 (**b**).

Figure 7 presents the correlation between the experimental moment, including the beam’s weight, and the corresponding strain measurements in both compressed concrete and tensioned GFRP reinforcement. For every experimental beam specimen, the reported GFRP strain represents the meaning of two discrete strain measurements taken at the midspan section. This averaging methodology was employed to account for potential local variations in strain distribution across the GFRP reinforcement. Table 7 shows a summary of recorded strains at 90% of ultimate moment and at failure for both top concrete fiber and GFRP bars. Cracks at locations of the strain gauges resulted in failure of these gauges even before the beam reached its ultimate capacity.

**Table 7:** Strains of the tested beam specimens.

Beam no.	Concrete strain		GFRP strain	
	ε_cu_ (µε)	ε_c−90%_ (µε)	ε_fu_ (µε)	ε_f−90%_ (µε)
2G10-2S10-C	− 6475	− 4942	19,687	16,250
2G10-2S10-E50	–*	− 4430	–*	17,607
2G10-2S10-E100	− 3929	− 3765	20,510	19,803
2G10-2S10-E150	–*	− 4810	–*	19,693
4G10-E100	− 5242	− 4780	18,788	16,485
4G10-EU50	− 4294	− 3898	20,513	18,438
2G10-2S10-EU50	–*	–*	22,075	19,140

ε_cu_ is the ultimate concrete strain; ε_c-90%_ is the ultimate concrete strain corresponding to 90% of the beam’s maximum capacity; ε_fu_ is the ultimate GFRP rebar strain; and ε_f-90%_ is the ultimate GFRP rebar strain corresponding to 90% of the beam’s maximum capacity.

*Strain gauges failed during loading.

The moment-strain relationship follows a similar three-stage progression as observed in the load-deflection response, for both hybrid and GFRP RC-beams, which reflects similar structural responses throughout the progressive structural deterioration phases from uncracked, cracked, and ultimately to failure stages. The strain recorded for GFRP bar at the ultimate load ranged from 70 to 83% with corresponding to the ultimate strain, which means no rupture in GFRP bars occurs at the ultimate load. Hybrid beams had greater stiffness than GFRP-RC beams up to the yielding. At approximately 75% of the ultimate moment, the hybrid-RC beam showed a noticeable reduction in stiffness compared to beams reinforced solely with GFRP bars, indicating a more rapid stiffness degradation beyond the steel yielding point.

### Moment-curvature relationship

**Fig. 8:**
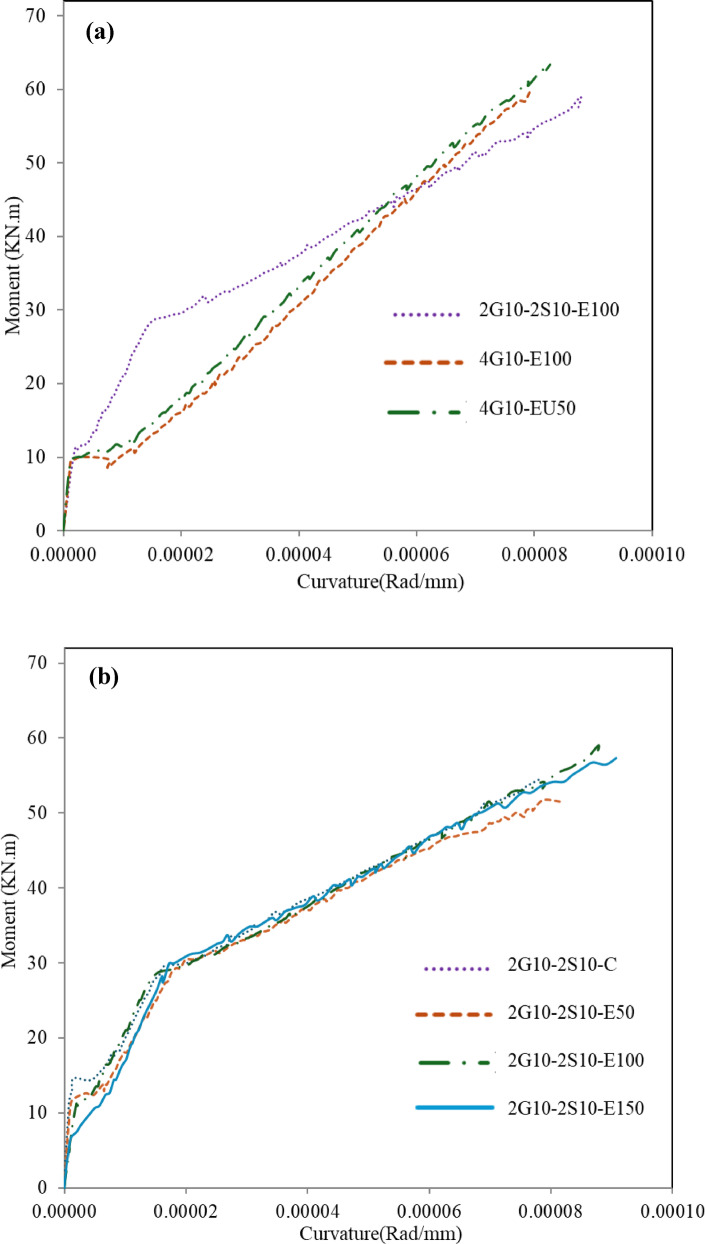
Moment versus curvature relationships; G1 (**a**); G2 (**b**).

Figure 8 indicates a relationship between the experimental moment and maximum curvature at the mid-span section. The section curvature is computed using the summation of concrete compressive strain and average tensile strain, both divided by effective beam depth. As mentioned previously, some strain gauges stopped reading before beams reached their ultimate capacity, and to ensure consistent comparative analysis across all beam specimens, curvature values were calculated at a standardized load level corresponding to 90% of each beam’s ultimate capacity. Comparing beams 4G10-E100 and 4G10-EU50, the curvature slightly decreased with using U-shape formwork. That’s due to the U-shape provides perfect lateral confinement, which carries part of the strain developed in the section instead of the reinforcement. With the increase in ECC thickness, no consistent trend can be established to predict curvature behavior. However, in general, it can be observed that the increase in ECC thickness resulted in higher curvature values up to the yielding. This behavior can be attributed to the formation of cracks within ECC, which leads to a redistribution of tensile stresses between the fibers and the reinforcement. Comparing beams 2G10-2S10-E100 and 4G10-E100 shows that GFRP-RC beams have higher curvature values up to the yielding. While after yielding, a rapid degradation in stiffness occurred, and hybrid beams exhibit a higher curvature value up to failure due to higher elongation occurring in steel bars after the yielding stage. It is important to note that the strain gauge attached to the top concrete fiber failed at the beginning of the test, and consequently, any results relying on its readings were no longer available.

### Evaluation of theoretical mid-span deflection

**Fig. 9:**
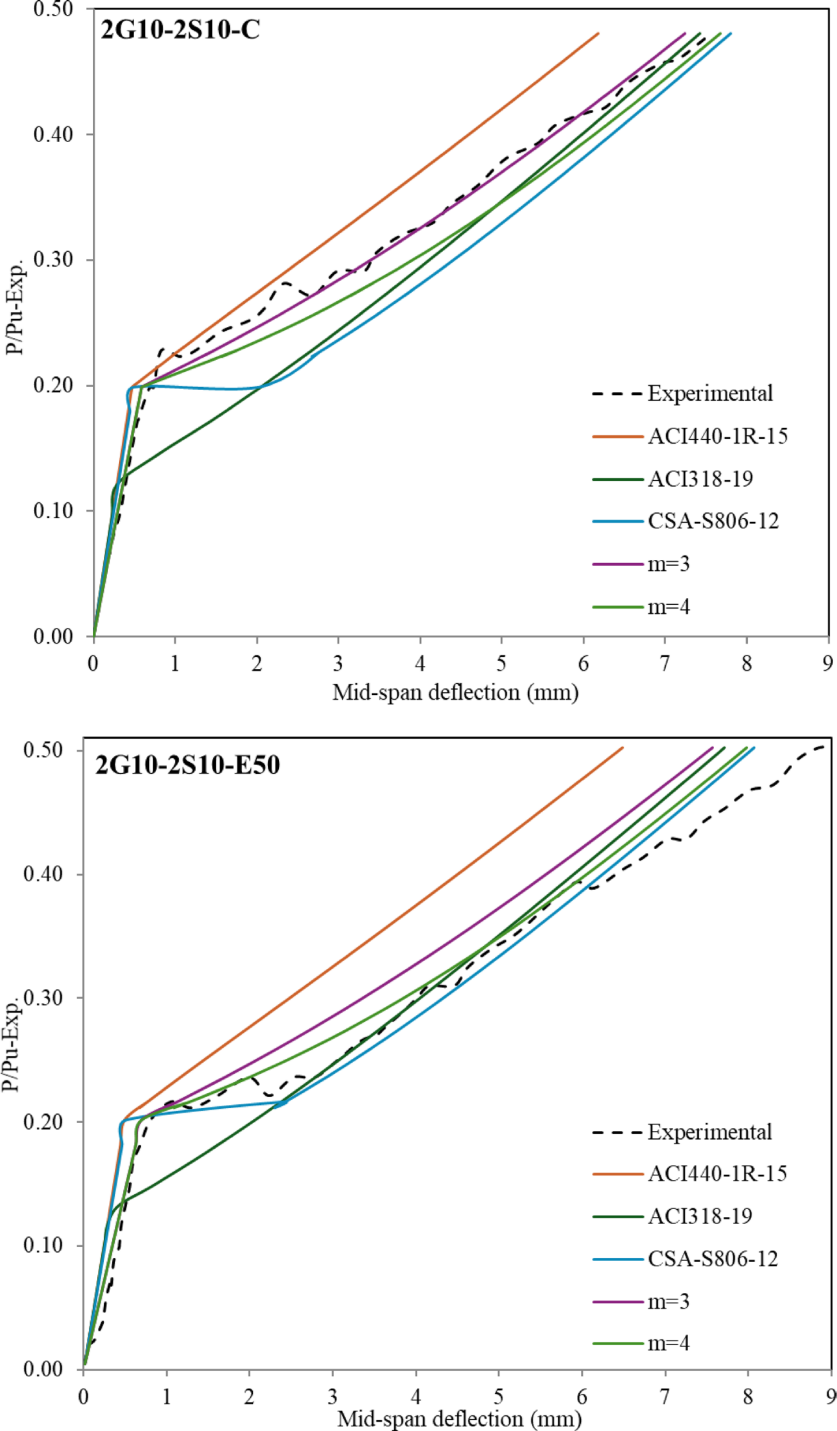

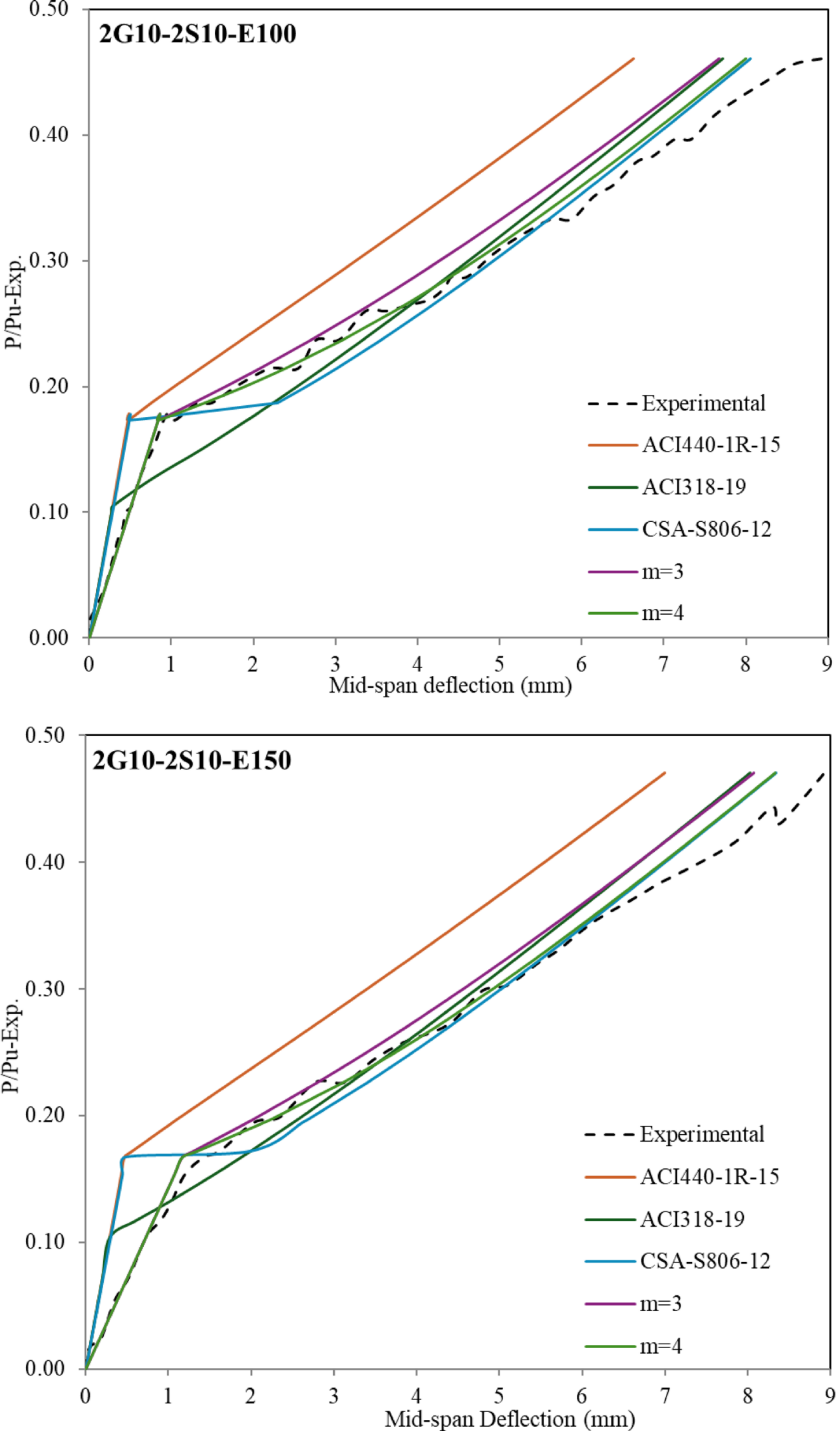

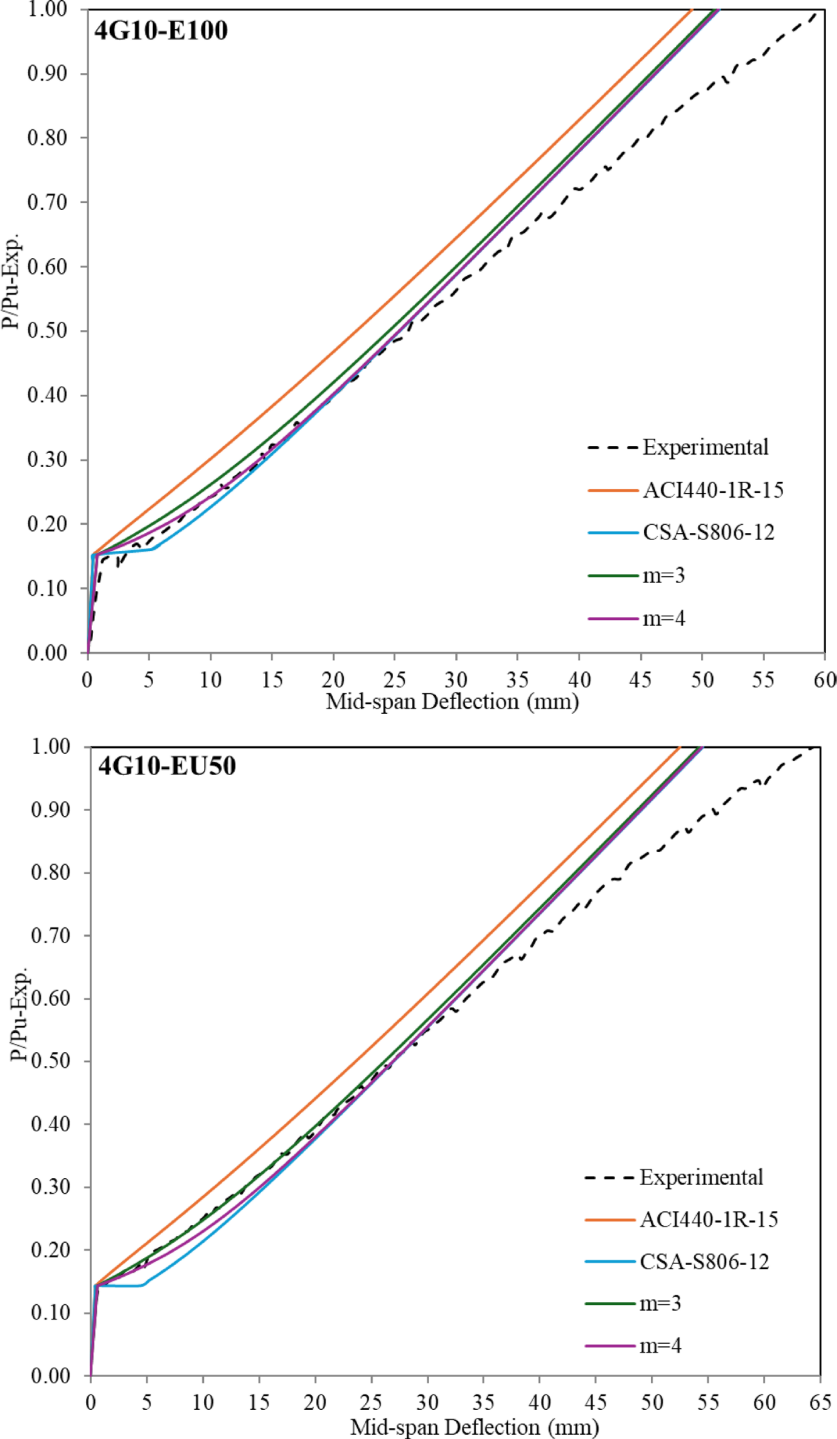

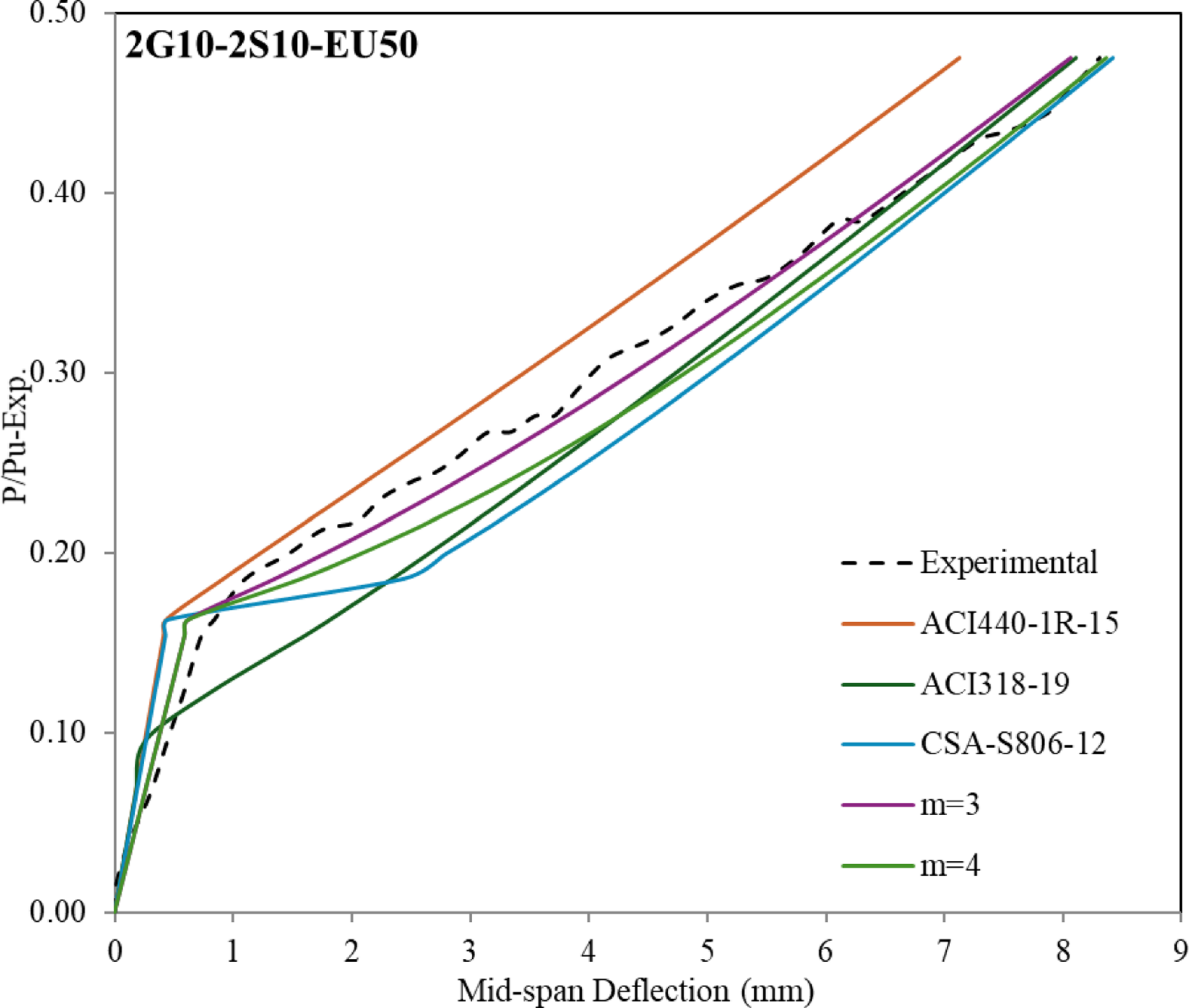
Comparison of mid span deflection between experimental and theoretical values up to yielding.

**Fig. 10:**
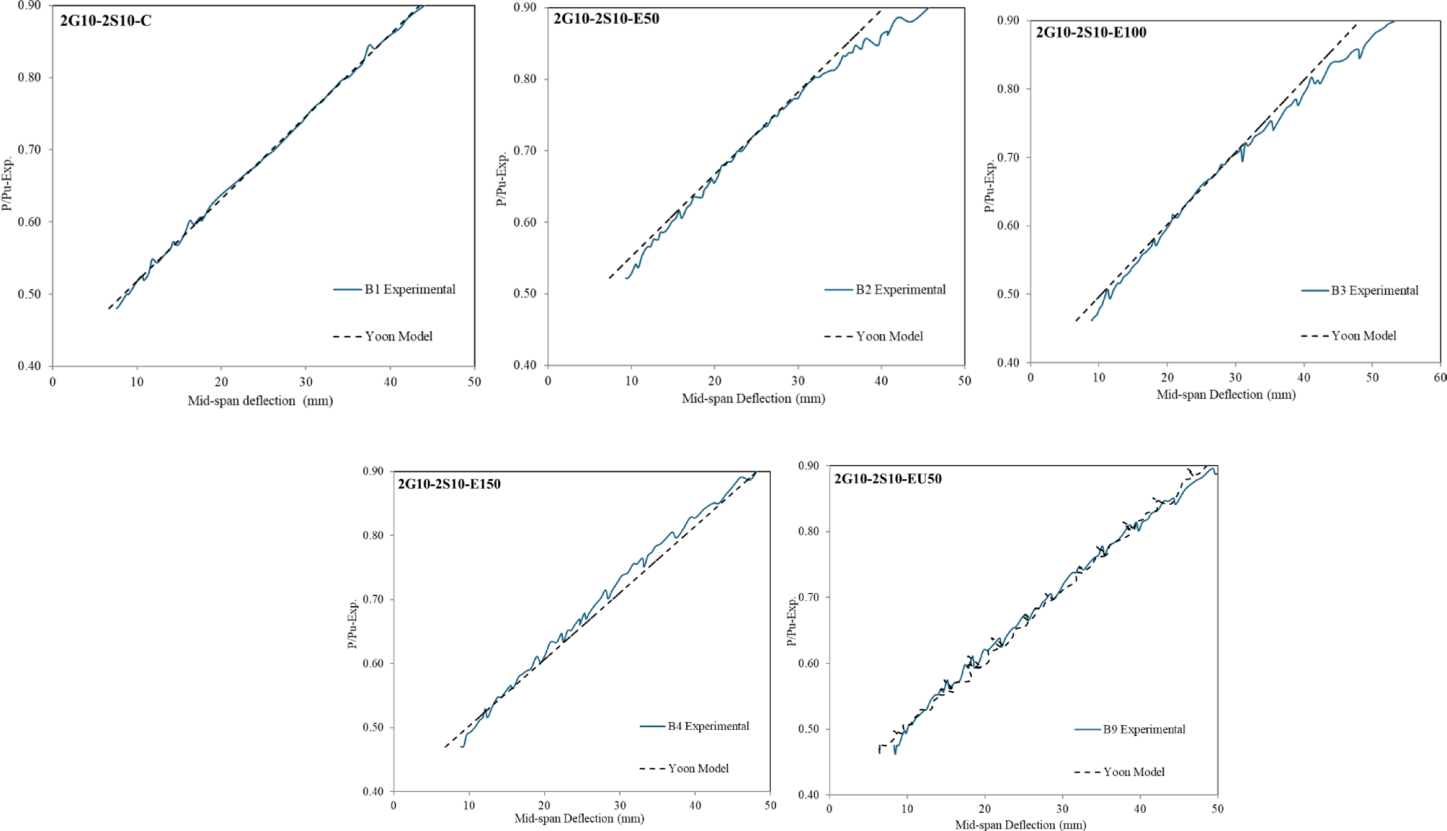
Comparison of mid span deflection between experimental and theoretical values for hybrid RC beams after yielding and up to 0.90 (P/Pu-exp).

The structural design of (steel-GFRP) hybrid-RC-beams is frequently governed by serviceability requirements due to the comparatively lower elastic modulus of GFRP reinforcement relative to steel. As a result, it’s important to determine the deflection behaviour in a hybrid system. The present investigation examines the relationship between load and deflection over the entire loading duration of the beam, with the analysis segmented into two distinct components for comprehensive assessment. The first segment examined the load-deflection behavior up to the yielding, according to the provisions of both the American and Canadian codes, which is represented in Fig. 9. The second segment focused on the behavior from the onset of yielding up to 90% of the ultimate load capacity, which is represented in Fig. 10.

### American codes (ACI)

According to ACI^4,43^, the maximum deflection at midspan for the four-point bending beam can be computed using Eq. (1).

1 $$\:{{\Delta\:}}_{\text{m}}=\:\frac{P\:a}{48\:{E}_{c}\:{I}_{e}\:}\:\left(3{L}^{2}\:-\:4{a}^{2}\right)$$

where Δ_m_ is peak mid-span deflection; *P* is the total acting load; *L* is the support span, which equals 2400 mm; $\:{E}_{c}\:$is the concrete modulus, taken as (4700 $\:\sqrt{{f}_{c}^{{\prime\:}}\:}$) as per (ACI); $\:{I}_{e}\:$is the effective moment of inertia; *a* is the shear span, which equals 900 mm.

Research by Bischoff and Scanlon (2007)^44^ demonstrated limitations in Branson’s formulation for calculating effective moments of inertia, particularly for members with steel reinforcement ratios below 1% or those incorporating FRP reinforcement. Consequently, they developed a modified expression suitable for flexural elements reinforced with either FRP or conventional steel bars. Current design practice follows ACI 440.1R-15^4^ for FRP-RC members and ACI 318 − 19^33^ for steel-RC elements when computing deflection behavior. Both methods were constructed based on Bischoff and Scanlon’s effective moment of inertia expression. Based on (ACI440.1R-15)^4^, the effective moment of inertia of pure FRP-RC-beams ($\:{I}_{e}$) calculated as presented in Eq. (2).

2 $$\:{I}_{e}=\:\frac{{I}_{cr}}{1-\:\gamma\:\:{\left(\:\frac{{M}_{cr}}{{M}_{a}}\:\right)}^{2}\left(\:1-\frac{{I}_{cr}}{{I}_{g}}\:\right)\:}\:\le\:{I}_{g}$$

where; $\:{M}_{a}$ is acting moment; $\:{I}_{cr}$ is transformed moment of inertia; $\:\gamma\:$ is a parameter that takes into account the member’s varying stiffness, and equal (1.72–0.72$\:\:\frac{{M}_{cr}}{{M}_{a}}\:$), $\:{M}_{cr}$ is defined as the first cracking moment, and $\:{I}_{g}$ calculated from Eq. (3).

Due to the different types of concrete used, the modulus of elasticity was determined using the ASTM C469 method. The modulus of elasticity of ECC (E_ECC_) equals 28 GPa, while the traditional concrete modulus (E_c_) equals 33 GPa. The gross concrete section’s moment of inertia (I_g_) was calculated due to the different types of material used in each tested beam, as illustrated in Eq. (3).

3 $$\:{I}_{g}=\frac{b\:{\left(h-{h}_{ec}\right)}^{3}}{12}+b\left(h-{h}_{ec}\right){\left({h}_{ec}+\frac{b\:\left(h-{h}_{ec}\right)}{2}-{y}_{t}\right)}^{2}+{n}_{ecc}[\:\frac{b\:{\left({h}_{ec}\right)}^{3}}{12}+b\:{h}_{ecc}{\left(\frac{{h}_{ec}}{2}-{y}_{t}\right)}^{2}]\:\:\:\:\:$$

where n_ecc_ is the ratio between (E_ECC_/E_c_), b is the section width, h is the section height, h_ec_ is ECC bottom layer thickness, and y_t_ is the tension face to centroid axis distance.

According to the code of ACI318-19^33^, the effective moment of inertia of pure steel-RC beams ($\:{I}_{e}$) calculated as $\:{I}_{g}$; If ($\:{M}_{a}$) is less than or equal to (2/3 $\:{M}_{cr}$), otherwise, if ($\:{M}_{a}$) is greater than (2/3 $\:{M}_{cr}$), $\:{I}_{e}$ can be determined as shown in Eq. (4).

4 $$\:{I}_{e}=\:\frac{{I}_{cr}}{1-\:\:{\left(\:\frac{{\left(\frac{2}{3}\right)M}_{cr}}{{M}_{a}}\:\right)}^{2}\left(\:1-\frac{{I}_{cr}}{{I}_{g}}\:\right)\:}$$

### Canadian code (CSA-S806-12)

According to CSA-S806-12^5^, the peak deflection at midspan for the four-point bending beam can be computed using Eqs. (5) and (6).

5 $$\:{{\Delta\:}}_{\text{m}}\:=\:\frac{P\:{L}^{3}}{48\:{E}_{c}\:{I}_{cr}}\:\left[3\left(\frac{a}{L}\right)-4{\left(\frac{a}{L}\right)}^{3}-8\left(\:1-\frac{{I}_{cr}}{{I}_{g}}\:\right){\left(\frac{{L}_{g}}{L}\right)}^{3}\right]$$

6 $$L_g = a\:\frac{{M}_{cr}}{{M}_{a}}$$

where Lg, in a simply supported beam, is the measured distance in mm from the support to the $\:{M}_{cr}\:$point, and $\:{E}_{c}\:$is taken as (4500 $\:\sqrt{{f}_{c}^{{\prime\:}}\:}$) as per (CSA). The moment of inertia of the cracked section ($\:{I}_{cr}$) calculated by Eqs. (7) and (8).

7 $$\:{I}_{cr}=\:\:\frac{b{d}^{3}}{3}{k}^{3}+\left({n}_{f}\:{A}_{f}+{n}_{s}\:{A}_{s}\right)\:{d}^{2}{\left(1-k\right)}^{2}$$

8 $$\:k=\sqrt{2\left({n}_{f}{\rho\:}_{f}+{n}_{s}{\rho\:}_{s}\right)+{\left({n}_{f}{\rho\:}_{f}+{n}_{s}{\rho\:}_{s}\right)}^{2}}-\left({n}_{f}{\rho\:}_{f}+{n}_{s}{\rho\:}_{s}\right)$$

where k is the dimensionless ratio of the neutral axis depth to the effective beam depth (c/d); n_s_ is the proportion of the elastic modulus of steel to that of concrete (Es/Ec); n_f_ is the proportion of the elastic modulus of GFRP to that of concrete (E_f_/E_c_).

The deflection at the mid span with a (P_y_/P_u−exp_) ratio up to the yielding stage is presented in Fig. 9. The theoretical deflection values using Eq. 1 and Eq. 2 (ACI440.1R-15 approach), the theoretical deflection using Eqs. (1) and (4) (ACI318-19 approach), and the theoretical deflection using Eq. (5) and (6) (CSA-S806-12 approach) which can be applied up to yielding only, both are given in Fig. 9. As shown in Fig. 9 and according to codes-based formulations, the predicted deflection before the cracking stage tends to be underestimated (less conservative) compared to experimental values. It was observed that increasing the thickness of the bottom ECC layer leads to a noticeable reduction in the overall stiffness. As a result, the discrepancy between the experimental and theoretical values becomes more pronounced. Accordingly, a correction factor, denoted by the symbol J, was proposed. This factor is multiplied by the moment of inertia values obtained from Eq. (3). The corresponding results are also presented in Fig. 9. It is important to emphasize that this proposed equation is derived based on a reinforcement ratio of (A_f_/A_s_) = 1.

9 $$J = 0.80-{\left(\frac{{h}_{ec}}{h}\right)}^{1.30}$$

The proposed equation has an acceptable prediction for hybrid RC-beams, as well as for U-shaped beams. However, it does not provide accurate results for beams incorporating a bottom ECC layer reinforced only with GFRP. Further studies are necessary to comprehensively study the behavior of such beams in this region when ECC is present. In general, the effective moment of inertia was found to decrease by approximately 0.40 to 0.80. Specifically, the beam with the maximum ECC thickness exhibited a reduction factor of approximately 0.40, whereas the beam without any ECC showed a factor close to 0.80. This reflects the impact of ECC on the stiffness of the section.

Theoretical deflection values calculated using ACI 440.1R-15 tend to underestimate the deflections of hybrid RC beams up to the yielding point when compared to experimental results. In contrast, for GFRP-RC beams, the predicted and measured deflections show good agreement up to failure. For hybrid RC beams, predictions based on ACI 318-19 and CSA codes align more closely with experimental observations, offering improved accuracy over ACI 440.1R-15. A modified equation for calculating the value of ($\:{I}_{e}$) by (ACI440.1R-15) has been proposed as presented in Eq. (10).

10 $$\:{I}_{e}=\:\frac{{I}_{cr}}{1-\:\gamma\:\:{\left(\:\frac{{M}_{cr}}{{M}_{a}}\:\right)}^{\text{m}}\left(\:1-\frac{{I}_{cr}}{{I}_{g}}\:\right)\:}\:\le\:{I}_{g}$$

The analysis incorporates the parameter m, which represents the tension stiffening effect. According to the provisions of the ACI440.1R-15 Code, when m equals 2, the section exhibits a well-developed tension stiffening behavior. In this study, the value of m varied between 3 and 4, and the corresponding effective moment of inertia ($\:{I}_{e}$) was recalculated accordingly. These recalculated values were then substituted into Eq. (1) to evaluate the structural response after cracking and up to the yielding stage. The deflection values calculated using Eqs. (9) and (10) were also included in Fig. 9 to clearly illustrate the differences and highlight the deviation between the analytical predictions and the experimental results. There is no universally fixed value for the parameter m (whether 3 or 4), but both significantly enhance accuracy compared to predictions by ACI 440.1R-15. Notably, m = 3 yielded better agreement for beams 2G10-2S10-C and 2G10-2S10-EU50, while m = 4 provided more accurate predictions for beams with ECC in the bottom layer or those reinforced solely with GFRP. These observations highlight the importance of tailoring m based on beam configuration to achieve closer alignment with experimental results.

The Bischoff model demonstrates limitations in predicting post-yield deflection behavior for hybrid reinforced beams, as the composite action between steel and GFRP reinforcement alters the structural response following steel yielding. This distinct phase requires alternative analytical approaches to accurately capture the nonlinear deformation characteristics. Thus, Yoon^45^ proposed, using Bischoff’s method, an effective moment of inertia after steel yields, the effective moment of inertia can be calculated by Eqs. (11), (12), and (13).

11 $$\:{I}_{e}=\:\frac{{I}_{cr}}{\frac{{I}_{cr}}{{I}_{y}}+\:\left(\:\frac{{M}_{y}}{{M}_{a}}\:\right)\:\left(\:1-\frac{{I}_{cr}}{{I}_{y}}\:\right)\:-\:{\left(\:\frac{{M}_{cr}}{{M}_{a}}\:\right)}^{2}\left(\:1-\frac{{I}_{cr}}{{I}_{g}}\:\right)\:}$$

12 $$\:{I}_{y}=\:\frac{b{d}^{3}}{3}{k}^{3}+\left({n}_{f}\:{A}_{f}\right){d}^{2}{\:\left(1-k\right)}^{2}$$

13 $$\:k=\:\sqrt{2\left({n}_{f}{\rho\:}_{f}\right)+{\left({n}_{f}{\rho\:}_{f}\right)}^{2}}-\left({n}_{f}{\rho\:}_{f}\right)$$

where ($\:{I}_{cr}$) is defined previously in Eq. (7); ($\:{I}_{y}$) is a representation of the cracked moment of inertia associated with the changed hybrid section following steel yielding, (M_y_) is the yielding moment measured from experimental work. Figure 10 represents a comparison between the theoretical deflection values using Eqs. (1) and 11 through Eq. (13) and the measured deflection values from experimental work after the yielding point and up to 0.90 (P/P_u−exp_) level for the hybrid RC-beam. The theoretical deflection values demonstrate a strong correlation with measured deflection values from experimental work for hybrid RC-beams with (A_f_/A_s_) equal 1.

### Ductility

The conventional ductility index quantifies a structural member’s deformation capacity through the ratio of ultimate deflection to yield deflection. This parameter fundamentally represents the system’s ability to undergo inelastic deformation and dissipate energy prior to reaching compressive failure in the concrete matrix.

Hybrid RC-beams’ ductility differs significantly from steel- or pure FRP-RC-beams. As a result, researchers have developed two principal methodologies for assessing ductility in these composite systems, namely: (1) energy-based approaches, and (2) deformation-based approaches.

**Fig. 11:**
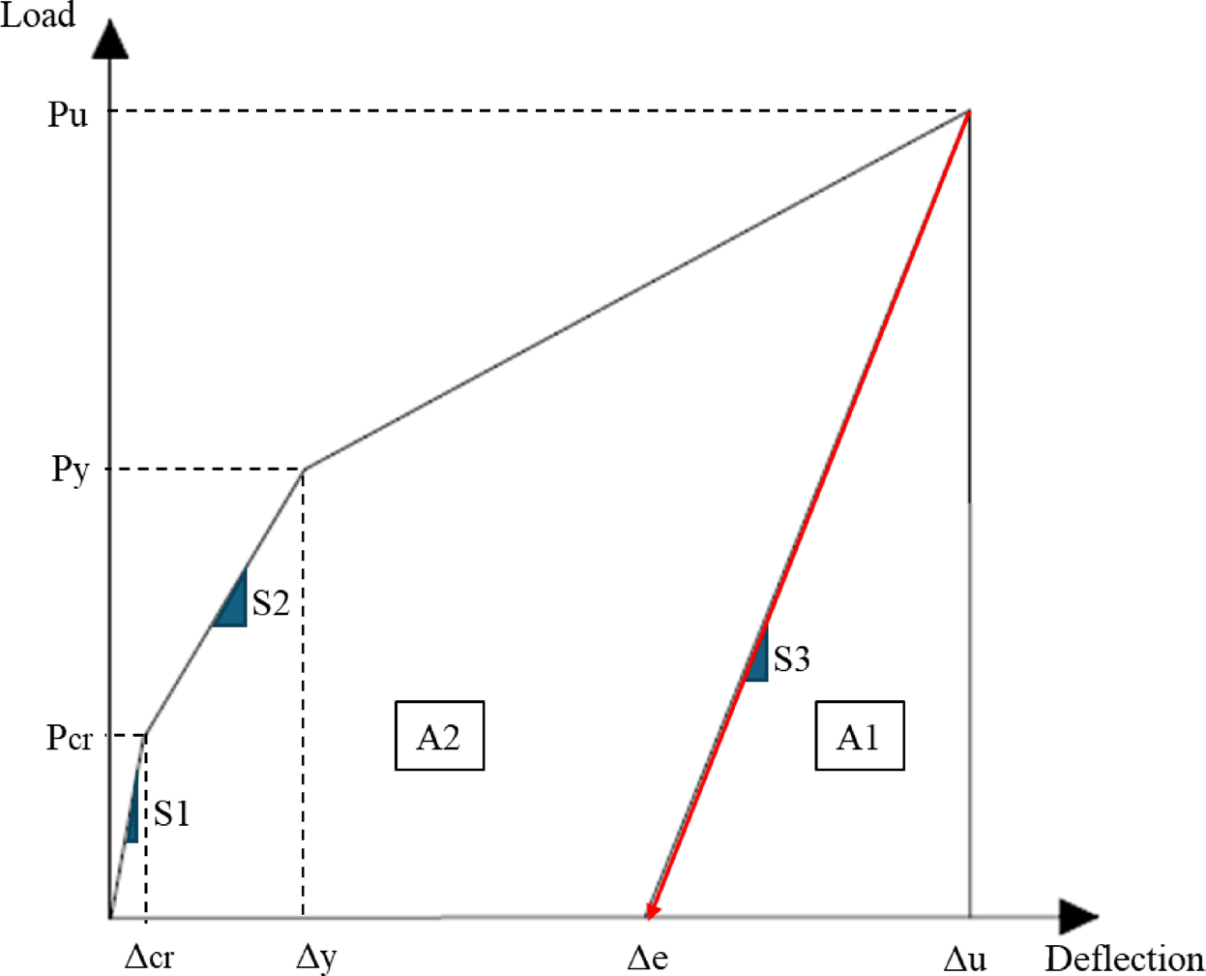
Energy-based approach for ductility computation.

In energy-based, ductility assessment quantifies a structural member’s capacity for energy absorption under flexural loading. Naaman and Jeong’s theory^46^ proposed that the ductility index (μ_e_) for FRP-RC-beams is derived from the ratio of total energy dissipation (including both elastic and inelastic components) to purely elastic energy capacity, as illustrated in Fig. 11, using Eq. (14).

14 $$\:{\mu\:}_{e}=\:\frac{1}{2}\left(\frac{{E}_{t}}{{E}_{e}}+1\right)$$

where the total energy absorption capacity ($\:{E}_{t}$) corresponds to the complete area beneath the load-deflection curve (A1 + A2), while the elastic energy component ($\:{E}_{e}$) is quantified by the triangular area under line S3 in the elastic region. As depicted in Fig. 11, line S3 extends from the ultimate load ($\:{P}_{u}$) to its intercept with the deflection axis (defining area A2). The slope of S3, calculable through Eq. (15), is geometrically determined by reference points S1, S2, $\:{P}_{\text{c}\text{r}}$ (cracking load), and $\:{P}_{\text{y}}$ (yield load). In GFRP-RC-beams, P_y_ is replaced with P_u_ in Eq. (15).

15 $$\:{S}_{3}=\:\frac{{P}_{cr}{S}_{1}+\left({P}_{y}-{P}_{cr}\right){S}_{2}}{{P}_{y}}$$

where S3 is the failure point’s unloading slope, which is also known as the average slope of the elastic region, S1 and S2. S1 denotes the loading curve’s initial slope, and S2 is the moment-deflection curve’s secant slope from the point of cracking until the yielding point. Table 8 presents a ductility index calculated by an energy-based approach.

As the thickness of the bottom ECC layer increases, both the total and elastic absorbed energy ($\:{E}_{t}$) and $\:({E}_{e}$), increase accordingly. Consequently, when calculating the ductility index ($\:{\mu\:}_{e}$) using Eq. (14), a noticeable reduction in ($\:{\mu\:}_{e}$) is observed, ranging from 7.50 to 40% compared to specimen 2G10-2S10-C. The use of the U-shaped ECC configuration generally leads to an increase in the ductility index ($\:{\mu\:}_{e}$), independent of the reinforcement type employed in the beam. For Hybrid RC-beams, this ratio increased by 8%, and for GFRP-RC-beams, this ratio increased by 22%. When comparing the influence of reinforcement type, hybrid RC beams demonstrated superior performance in terms of total absorbed energy and ductility index relative to the GFRP-RC beam.

Jeager’s experimental investigation^47^ systematically compared the deformation capacity of FRP-reinforced and steel-reinforced beams through a deformation-based methodology. The study established that the deformability index ($\:{\mu\:}_{E,\varDelta\:}$) for both beam types can be quantitatively expressed using Eq. (16). To maintain consistency in the analysis, the ductility of all tested beams was calculated at 90% of their ultimate capacity, as some strain gauges failed to record data prior to reaching maximum load.

16 $$\:{\mu\:}_{E,\varDelta\:}=\:\frac{{\phi\:}_{u-90\%}}{{\phi\:}_{0.001}}\frac{{M}_{u-90\%}}{{M}_{0.001}}$$

where $\:{\mu\:}_{E,\varDelta\:}$ is the deformability index; $\:{M}_{u-90\%}$ is the moment at 90% of ultimate capacity; $\:{\phi\:}_{u-90\%}$ is the curvature at 90% of ultimate capacity; and $\:{M}_{0.001}$ and $\:{\phi\:}_{0.001}$ are the moment and curvature, at a service limit state, respectively, with the corresponding concrete strain equal to 0.001. Also, the values of moment and curvature at strain equal 0.001 were replaced with moment and curvature values at yielding at the previous Eq. (16), and the deformability index was recalculated. Table 9 presents a ductility index calculated by a deformability-based approach. There is no universal standard for quantifying the variations observed in beam behavior; however, it was generally noted that increasing the thickness of the bottom ECC layer leads to an increase in deformability index, as calculated based on the yielding strain approach. A similar trend was recorded for RC-beams exclusively with GFRP bars, where the deformability index showed a noticeable increase compared to hybrid-RC beams.

**Table 8:** Ductility index calculated by Energy-Based approach.

No.	*P*_cr_ (KN)	*P*_y_ (KN)	*P*_u_ (KN)	Δ_cr_ (mm)	Δ_y_ (mm)	Δ_u_ (mm)	Δ_e_ (mm)	E_t_ (KN.mm)	E_e_ (KN.mm)	µ_e_
2G10-2S10-C	26.24	63.36	131.84	0.69	7.56	55.78	48.81	5023	459	5.97
2G10-2S10-E50	26.24	67.84	129.92	0.8	9.29	59.73	51.45	5397	538	5.52
2G10-2S10-E100	24.32	64.64	140.16	1.05	8.93	64.49	52.72	6053	825	4.17
2G10-2S10-E150	23.68	66.56	141.44	1.43	8.92	67.19	52.42	6415	1044	3.57
4G10-E100	22.4	–	146.56	1.96	-	59.51	18.51	4884	3005	1.31
4G10-EU50	21.76	–	155.52	1.04	-	64.37	31.59	5625	2549	1.60
2G10-2S10-EU50	23.04	67.2	141.44	0.84	8.31	62.4	51.76	5989	753	4.48

**Table 9:** Ductility index calculated by deformability-based approach.

Beam No.	φ_u−90%_ (10^− 5^/mm)	M_u−90%_ (KN.m)	φ_0.001_ (10^− 5^/mm)	M_0.001_ (KN.m)	φ_y_ (10^− 5^/mm)	M_y_ (KN.m)	Deformability index	
							1000 (µε)	Yielding strain
2G10-2S10-C	7.85	54.63	1.47	26.11	1.74	29.57	11.16	8.34
2G10-2S10-E50	8.16	52.59	1.42	25.23	1.67	31.57	11.97	8.16
2G10-2S10-E100	8.73	58.35	2.23	31.57	1.76	30.12	7.22	9.62
2G10-2S10-E150	9.08	58.35	1.39	24.65	1.73	30.99	15.46	9.90
4G10-E100	7.88	59.50	1.69	15.15	–	–	18.36	–
4G10-EU50	8.27	64.40	2.34	21.49	–	–	10.58	–
2G10-2S10-EU50	–	–	–	–	–	–	–	–

## Conclusion

The combined use of ECC materials and hybrid (steel-GFRP) bars in concrete beams significantly improves durability and prevents reinforcement corrosion. This research evaluates both the serviceability and ductility behaviour of partially composed ECC RC-beams. Seven beams were designed with the aim of studying the behaviour of partial precast ECC concrete in the tension zone of the tested beams. Multiple factors, including reinforcement type and ECC configuration as a bottom layer with different thickness or U-shape formwork, were examined. Two concentrated loads were applied incrementally until beam failure. The experimental results include cracks distribution, load strain, and moment curvature relation. The validity of different codes’ equations to calculate deflection and assess of ductility of the beams was checked.

These findings could contribute to safer, corrosion-resistant infrastructure in coastal areas. Moreover, the results highlight the potential use of ECC as a material for precast components, particularly in configurations where ECC is cast in a U-shape and later filled on-site with traditional concrete or grout. Similarly, specimens with varying ECC thicknesses suggest that ECC can serve as a permanent formwork, reducing the need for full-length bottom shuttering and requiring only temporary propping. The experimental and analytical findings collectively led to the following conclusions:

- All GFRP-RC beams exhibited brittle failure once the concrete reached its ultimate strength, whereas the hybrid-RC beams demonstrated ductile behavior beyond the concrete’s ultimate strength.
- Using U-shaped ECC concrete instead of a rectangular bottom layer (with the same volume) enhances post-cracking stiffness and ultimate strength, while also improving interface shear transfer. This configuration effectively prevents bond failure at the interface and ensures composite action of the beam components up to failure.
- Increasing the thickness of ECC concrete in tension layer affected each of the following; (a) the distribution of vertical cracks increased along the beam length as the thickness of ECC increases; (b) noticeable reduction in the overall stiffness before cracking; (c) the yielding and the ultimate and deflection values increased; (d) the total and elastic absorbed energy ($\:{E}_{t}$) and $\:({E}_{e}$) increased.
- The deflection values calculated using ACI 318-19 and CSA S806-12 equations showed good correlation with the experimentally measured deflections for all tested beams. In contrast, the predictions obtained using the ACI 440.1R-15 equations did not accurately capture the deflection behavior across all specimens. Additionally, Yoon’s model demonstrated a reliable capability to predict the deflection of hybrid beams beyond the yielding point.
- Modified moment of inertia using the suggested factor (m), whether it is equal to 3 or 4, gets better results compared to those provided by ACI 440.1R-15.
- The use of U-shaped ECC configuration leads to an increase in the ductility index ($\:{\mu\:}_{e}$), independent of the reinforcement type employed in the beam.
- Hybrid RC-beams demonstrated superior performance in terms of total absorbed energy and ductility index relative to the GFRP-RC-beam.

## Data Availability

The research data underpinning these findings are presented in this article, with additional information available from the lead investigator upon reasonable request. The corresponding author should be contacted for any required data.

## Abbreviations

FRP: Fiber reinforced polymer
GFRP: Glass-fiber-reinforced-polymer
ECC: Engineered cementitious composite
RC: Reinforced concrete
PE: Polyethylene fiber
PVA: Polyvinyl alcohol fiber
*b*: Beam width
d: Beam depth
A_s_: Steel reinforcement
A_f_: GFRP reinforcement
h_ec_: Height of ECC concrete
h: Beam height
L: Support length
I_e_: Effective moment of inertia
Δ_m_: Mid-span deflection
μ_e_: Energy ductility index
μ_E,Δ_: Deformability ductility index
